# Effect of ultra-processed foods consumption on glycemic control and gestational weight gain in pregnant with pregestational diabetes mellitus using carbohydrate counting

**DOI:** 10.7717/peerj.10514

**Published:** 2021-02-01

**Authors:** Carolina F. M. Silva, Claudia Saunders, Wilza Peres, Bárbara Folino, Taiana Kamel, Mayara Silva dos Santos, Patrícia Padilha

**Affiliations:** 1 Institute of Nutrition Josué de Castro, Federal University of Rio de Janeiro, Rio de Janeiro, Rio de Janeiro, Brazil; 2 Veiga de Almeida University (UVA), Rio de Janeiro, Brazil

**Keywords:** Maternal nutrition, Prenatal care, Diabetes mellitus, Blood glucose, Weight gain

## Abstract

**Aims:**

The aims were to evaluate the consumption of ultra-processed foods by pregnant women with pre-existing diabetes mellitus (DM) using the carbohydrate counting method, in addition to investigating the association with total gestational weight gain and glycemic control.

**Methods:**

A cohort study of adult Brazilian pregnant women with pre-existing DM. Dietary intake was evaluated adopting the *NOVA* classification to identify the reported consumption of ultra-processed foods. Weight was measured at all consultations and laboratory tests were evaluated at each gestational trimester. Multivariate linear regression was used in the analysis.

**Results:**

Pregnant women (*n* = 42) presented mean total gestational weight gain of 12.02 ± 4.8 kg, 65.8% of them with inadequate weight gain. Daily consumption of ultra-processed foods was 272.37 ± 170.55 kcal. The increase of every 1 kcal in the calorie intake from ultra-processed foods in the third trimester increased glycated hemoglobin by 0.007% (β = 0.007, *p* = 0.025), raised 1-h postprandial glucose by 0.14 mg/dL (β = 0.143, *p* = 0.011), and added 0.11 kg to total gestational weight gain (β = 0.11, *p* = 0.006).

**Conclusion:**

Ultra-processed food consumption influenced glycemic control and total gestational weight gain in pregnant women with DM.

## Introduction

In pregnant women with pre-existing diabetes mellitus (DM), physiological changes in insulin sensitivity lead to substantially higher rates of perinatal complications than seen amongst the general population (American Diabetes Association, 2020; Egan, Murphy & Dunne, 2015).

High levels of glycemia during pregnancy may be associated, among other complications, to excessive fetal weight and length, neonatal hypoglycemia and other metabolic disorders, and an increased risk of obesity and type 2 DM in adulthood for the newborns (National Institute for Health and Care Excellence, 2015; Metzger et al., 2008). Pregnant women with DM have an increased risk of hypertensive pregnancy syndromes, premature delivery, cesarean delivery, and obstetric trauma (Egan, Murphy & Dunne, 2015; Holmes et al., 2011).

Moreover, as gestation is a time when women naturally gain weight, it may itself predispose to obesity, due to an increase in postpartum weight retention, if this gain is excessive (Rong et al., 2015).

Healthy eating is one of the key factors to meet increased maternal nutritional demand and promote adequate fetal growth and development, and represents one of the pillars of adequate glycemic control (American Diabetes Association, 2020; Procter & Campbell, 2014). Since the Diabetes Control and Complications Trial (The DCCT Research Group, 1993), carbohydrate counting has been described as an important dietary planning method for people with diabetes, associated with reduced glycated hemoglobin (HbA1c) and increased quality of life (Ulahannan, Ross & Davies, 2007; Laurenzi et al., 2011). This method is characterized by greater autonomy and flexibility of food choices, with regular nutritional education guidelines and actions being essential to ensure the quality of the diet (Souto & Rosado, 2010; Kawamura, 2007).

In 2014, the Brazilian government’s dietary recommendations for the general population (Food guide for the Brazilian population) drew attention to the relationship between food choices and health. The dietary guidelines are based on the *NOVA* classification, which categorizes foods according to the type of processing to which they are submitted before their acquisition, preparation, and consumption. In the guide, the consumption of fresh or minimally processed foods is encouraged, and the consumption of ultra-processed foods is proscribed (Ministério da Saúde, 2014).

In Brazil, the increase in the consumption of ultra-processed foods is higher than in developed countries (Monteiro et al., 2013), and is significantly higher even in the diets of pregnant women (Alves-Santos et al., 2016). Recent evidence points to the negative effects of the consumption of ultra-processed foods on the lipid profile, weight gain, and prevalence of metabolic syndrome in different populations (Mozaffarian et al., 2011; Rauber et al., 2015; Rohatgi et al., 2017). However, no data regarding pregnant women with pre-existing DM is to be found in the scientific literature or on the potential risks to the mother and child. The present study aims to evaluate the consumption of ultra-processed foods by pregnant women with pre-existing DM and its effect on glycemic control outcomes and total gestational weight gain using carbohydrate counting.

## Materials and Methods

### Study design

This is a cohort study carried out with pregnant women with pre-existing DM who attended prenatal care services and received nutritional follow-up care at a public maternity hospital in the city of Rio de Janeiro. It is part of a larger study entitled “Theoretical and Practical Contributions to the Prenatal Care of Diabetic Pregnant Women” (ReBEC RBR-524z9n), developed under the responsibility of the Maternal and Infant Health Research Group at the Federal University of Rio de Janeiro (INJC/UFRJ).

The maternity unit was selected because it provides free care for pregnant women with the same characteristics as those who attend other clinics in the city of Rio de Janeiro, according to the variables maternal age and number of prenatal care consultations. Data were collected between July 2011 and October 2014.

### Research subjects

The study population consisted of pregnant women with pre-existing DM. The inclusion criteria adopted were: age ≥ 20 years at conception; single-fetus pregnancy; attended prenatal care; and received nutritional guidance based on carbohydrate counting. The women for whom there was no information about their dietary intake, who had a miscarriage, and who were diagnosed with a chronic disease other than obesity were excluded from the study.

### Dietary planning

Total energy value required for each pregnant woman was calculated according to weekly and total recommended gestational weight gain (up to the 40th gestational week), based on their initial nutritional status, according to the *Institute of Medicine* recommendations (Saunders, Bessa & Padilha, 2010; Kawamura, 2009) and individualized nutritional guidance, based on carbohydrate counting (Sociedade Brasileira de Diabetes, 2019).

As prescribed by this method, total carbohydrates in grams or number of substitutions per meal were counted, which covered the daily recommended allowance of the macronutrient. A list of carbohydrate equivalents was adopted, in which the foods were grouped so that each portion of the food chosen by the participant corresponded to 15 g carbohydrate, classifying them into categories (food groups) (Sociedade Brasileira de Diabetes, 2019). Alongside the autonomy they gained by following this method, the pregnant women were also advised about the importance of making appropriate nutritional choices to better promote the desired gestational outcomes.

The prescribed carbohydrate distribution varied between 45% and 55% of the total energy value (TEV), defined after a detailed nutritional assessment in a consultation with the nutritionist, as well as 25–35% fat and 15–20% proteins of the TEV (Sociedade Brasileira de Diabetes, 2019; American Diabetes Association, 2004).

### Research instrument

Information on food consumption was gathered and anthropometric, socio-demographic, laboratory, and obstetric data were collected using a structured service protocol that was completed in face-to-face interviews, and by consulting medical records.

#### Evaluation of food consumption

A semi-quantitative food frequency questionnaire was adopted to measure dietary intake and was administered by a nutritionist in the second and third trimesters. A table of home measurements was used to quantify the foods consumed (in grams and milliliters) (Pinheiro et al., 2005). The frequencies reported were transformed into daily frequencies. The chemical composition of the foods in energy (in kcal) was calculated according to the Brazilian Food Composition Table (Center for Studies and Research in Food (NEPA- UNICAMP), 2011).

Ultra-processed foods were identified according to the *NOVA* classification proposed in the *Food guide for the Brazilian Population* (Ministério da Saúde, 2014), and were quantified according to their energy value (EV) and percentage contribution to total daily energy consumption reported by the women.

Ultra-processed foods are products whose manufacture involves various processing steps and techniques and various ingredients, many of them exclusively for industrial use (Ministério da Saúde, 2014).

#### Anthropometric evaluation

The women’s weight and height were measured and used to calculate their pre-gestational body mass index (BMI) (in kg/m^2^). Pre-gestational weight was the self-reported or measured weight up to the 13th gestational week (in kg). Weight was measured at all visits and shortly before delivery.

#### Socio-demographic evaluation

The variables were: maternal age (in years); skin color by self-classification (white/black/brown/yellow); marital status (single/stable relationship); family income per capita (in reais); schooling (complete); housing sanitary conditions (adequate/inadequate); social habits (smoking/drinking/neither); housing area; and parity (nulliparous/multiparous).

#### Obstetric evaluation

Data on the number of deliveries, intergestational interval (in months), pre-delivery weight (in kg), and gestational age at delivery (in weeks, as measured by ultrasound) were collected.

#### Laboratory evaluation

Fasting and 1-h postprandial glucose (in mg/dL) and HbA1c (in %) were analyzed. Glycemic control was classified as: good, when fasting glucose <95mg/dl and 1 h postprandial <140 mg/dL, or 2 h postprandial <120 mg/dL in the period between the previous and present consultations; and bad if one of the parameters exceeded the cutoff points (American Diabetes Association, 2020). The prescribed insulin doses (in IU/kg) were also considered.

### Variables of interest

The dependent variables were glycemic control, evaluated by HbA1c (in %), 1-h postprandial glucose (in mg/dL), and total gestational weight gain (in kg). The variables for characterizing the sample were anthropometric, socio-demographic, and obstetric. The independent variable investigated was the consumption of ultra-processed foods in the second and third trimesters (in kcal and % contribution to total daily energy intake).

### Statistical analysis

An exploratory analysis of the data was performed and the normality of the outcomes was tested by the Kolmogorov–Smirnov test.

The bivariate linear regression was used to identify the independent variables to be tested in the multivariate linear model, with *p* < 0.20. Next, multivariate linear regression models were tested by the stepwise method, including variables with *p* < 0.20 in the bivariate analysis and *p* < 0.05 as the criterion of permanence in the final model, to identify the variables. Variables and the adjusted β coefficients were estimated, with their respective 95% confidence intervals.

In all the analyses, a significance level of 5% was adopted; the statistical package used was SPSS for Windows, version 21.0.

### Sample size

Considering that approximately 40 pregnant women with DM attend the study’s maternity hospital, per year, a minimum sample size of 40 pregnant women, was estimated as the sample of the convenience study.

### Ethical considerations

Each study participant signed a consent form and the study was approved by the Research Ethics Committee of MaternidadeEscola/UFRJ (Maternity School/Federal University of Rio de Janeiro; CAAE—0017.0.361.361-10).

## Results

**Figure 1:**
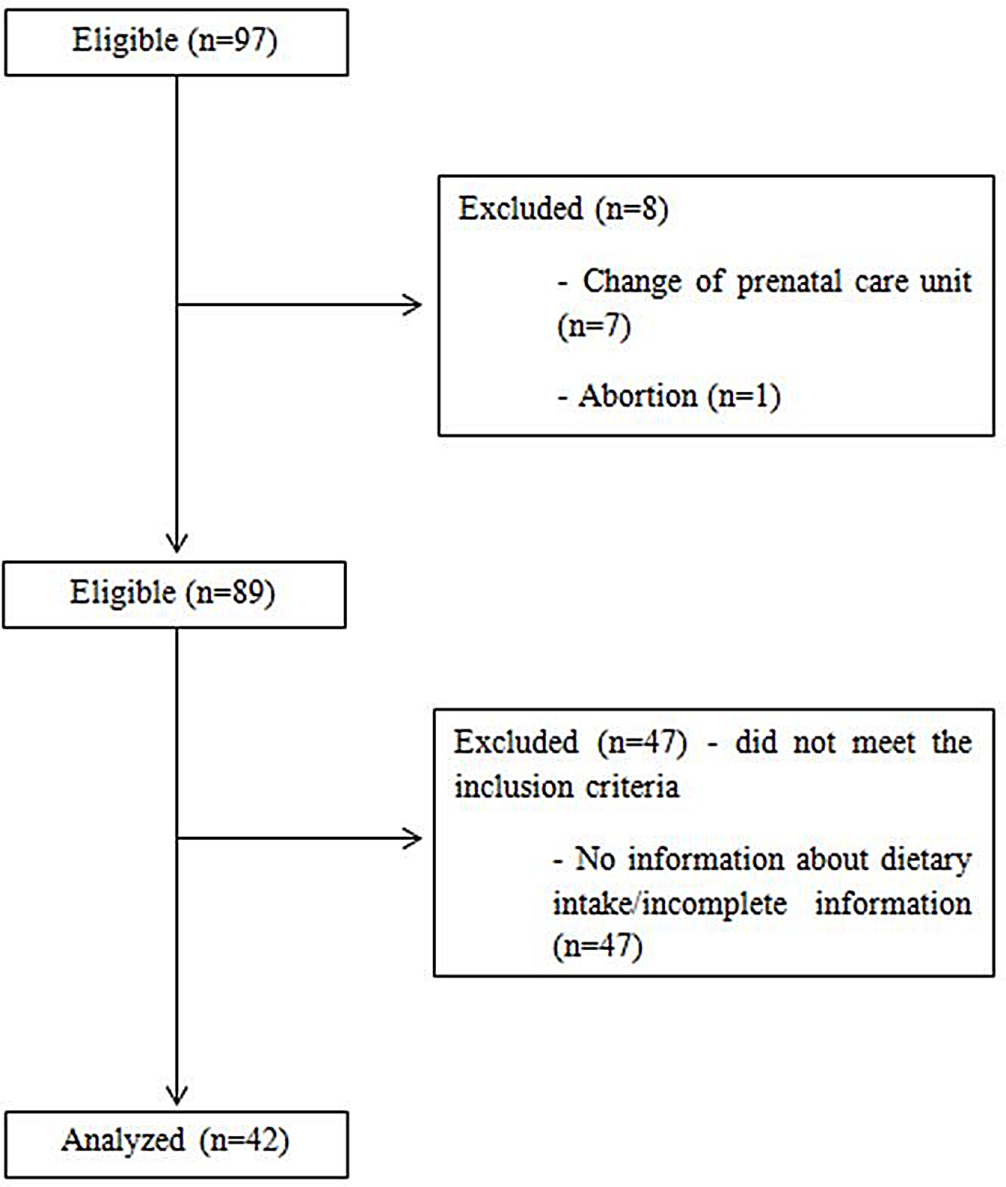
Flow chart of the sample of pregnant women with pre-existing diabetes mellitus selected for the study.

Figure 1 shows the flow chart of the sample of 42 pregnant women were considered included in the study because they met the inclusion criteria. Regarding the general characteristics of the sample, the women were mostly multiparous, with an average gap between pregnancies of approximately 75.6 months (±49.7). Most of the women had brown skin (38.1%, *n* = 16), were in a stable relationship (76.2%, *n* = 32), had completed their secondary education (59.5%, *n* = 25), did not smoke or drink alcohol (95.2%, *n* = 40), lived in the north and west area of Rio de Janeiro (52.4%, *n* = 22), and classified their housing conditions as sanitary (90.5%, *n* = 38).The pregnant’s mean age was 31.5 ± 5.8 years.

Regarding the anthropometric characteristics, as descripted in Table 1, 64.3% (*n* = 27) of the pregnant women were overweight or obese. Total gestational weight gain was 12.02 ± 4.8 kg on average, and more than half (51.2%) gained more weight than was recommended for their pre-gestational BMI.

**Table 1:** Characteristics of pregnant women with pre-existing diabetes mellitus treated at a public maternity hospital in Rio de Janeiro, Brazil, 2011–2014 (*n* = 42).

Continuous variables	Mean (SD)
Age, in years (*n* = 42)	31.52 (5.84)
EV consumed in the second trimester, in kcal (*n* = 42)	1,874.38 (368.61)
EV consumed in the third trimester, in kcal (*n* = 42)	1,797.24 (393.71)
EV from UPF in the second trimester, in kcal (*n* = 42)	317.29 (187.28)
EV from UPF in the third trimester, in kcal (*n* = 42)	272.37 (170.55)
Gestational weight gain, in kg (*n* = 41)	12.02 (4.8)
Weight gain in the first trimester, in kg (*n* = 42)	2.5 (2.56)
Weight gain in the second trimester, in kg (*n* = 42)	5.49 (2.98)
Weight gain in the third trimester, in kg (*n* = 41)	4.02 (2.14)
Categorical variables	n (%)
Gestational age at delivery (*n* = 41)	
<37 weeks	11 (26.8)
≥37 weeks	30 (73.2)
Parity (*n* = 42)	
Nulliparous	15 (35.7)
Multiparous	27 (64.3)
Pre-gestational nutritional status, according to BMI (*n* = 42)	
Underweight	1 (2.4)
Normal weight	14 (33.3)
Overweight	12 (28.6)
Obesity	15 (35.7)
Adequacy of weight gain^a^(*n* = 41)	
Below	6 (14.6)
Adequate	14 (34.2)
Above	21 (51.2)

**Notes:**

a According to IOM, 2009.

SD, standard deviation; EV, energetic value; UPF, ultra processed food; BMI, body mass index.

As shown in the same table the mean total calorie intake reported by the women was 1,874.38 ± 368.61 kcal in the second trimester and 1,797.24 ± 393.71 kcal in the third, with no statistical difference between them (*p* = 0.471). Meanwhile, the intake of ultra-processed foods in the second and third trimesters was 317.29 ± 187.28 kcal and 272.37 ± 170.55 kcal, respectively, representing 16.9 ± 7.7% and 15.2 ± 10% of average daily energy consumption.

Regarding the type of DM, 45.2% had type 1 and 47.6% had type 2, with an average diagnosis time of 8.5 ± 7.03 years. All of them used insulin during gestation, with mean doses of 0.84 ± 0.35 IU/kg in the first trimester, 1.02 ± 0.41 IU/kg in the second trimester, and 1.15 ± 0.5 IU/kg in the third trimester.

As shown in Table 2, there was a reduction in the subjects’ fasting and 1-h postprandial glucose levels and glycated hemoglobin levels over the gestation period. There was a significant improvement in adequacy of glycemic control as measured by fasting glucose over the trimesters, which was found to be 3.2%, 23.1%, and 33.3% in the first, second, and third trimesters, respectively (*p* = 0.03, *n* = 31).

**Table 2:** Glycemic control of pregnant women with pre-existing diabetes mellitus attended at a public maternity hospital in Rio de Janeiro, Brazil, 2011–2014 (*n* = 42).

Continuous variables	Mean (SD)
Fasting blood glucose (mg/dL)	
First trimester (*n* = 31)	145.04 (58.48)
Second trimester (*n* = 39)	121.76 (39.8)
Third trimester (*n* = 39)	102.25 (46.3)
Blood glucose 1 h postprandial (mg/dL)	
First trimester (*n* = 25)	213.95 (64.49)
Second trimester (*n* = 37)	177.82 (53.78)
Third trimester (*n* = 39)	154.34 (60.7)
Glycated hemoglobin (%)	
First trimester (*n* = 18)	8.2 (1,7)
Second trimester (*n* = 24)	6.7 (1,4)
Third trimester (*n* = 12)	6.7 (1,5)
Categorical variables	n (%)
Glycemic control^a^ first trimester (*n* = 31)	
Adequate	1 (3.2)
Inadequate	30 (96.8)
Glycemic control^a^ second trimester (*n* = 39)	
Adequate	9 (23.1)
Inadequate	30 (76.9)
Glycemic control^a^ third trimester (*n* = 39)	
Adequate	13 (33.3)
Inadequate	26 (66.7)

**Notes:**

a according to ADA, 2018.

SD, standard deviation.

In the bivariate linear regression, the variables related (*p* < 0.20) to HbA1c concentration in the third trimester were: EV consumed from ultra-processed foods in the third trimester of gestation (*p* = 0.025) and number of prenatal consultations (*p* = 0.148). Although the *p*-value was >0.20 for pre-gestational BMI (*p* = 0.723) and maternal age (*p* = 0.915) in the bivariate analysis, they were tested in the final model by biological plausibility. Table 3 shows the final model adjusted for these variables: every 1 kcal increase in EV from ultra-processed foods increased HbA1c by 0.007% in the third trimester (β = 0.007, *p* = 0.025). The concentration of HbA1c in the second trimester of gestation was not influenced by the consumption of ultra-processed foods (*p* = 0.923).

**Table 3:** Linear regression of the factors associated with glycated hemoglobin concentration and total weight gain of pregnant women with pre-existing diabetes mellitus. Rio de Janeiro, Brazil, 2011–2014 (*n* = 42).

Variables	β_ajusted_	IC 95%	*p*
Glycated hemoglobin (%)			
Maternal age (years)	−0.082	[−0.264 to 0.99]	0.319
EV from UPF in third trimester (kcal)	0.007	[0.001–0.013]	0.025
Numberofprenatalconsultations	−0.141	[−0.446 to 0.164]	0.310
Pre-gestational BMI (kg/m^2^)	−0.118	[−0.072 to 0.309]	0.186
Glicemia pós-prandial 1 h no 3° trimestre (mg/dL)			
Maternal age (years)	−3.360	[−6.953 to 0.233]	0.066
EV from UPF in third trimester (kcal)	0.143	[0.034–0.251]	0.011
Numberofprenatalconsultations	−8.002	[−15.395 to −0.609]	0.035
Number of consultations with nutritionist	7.522	[−6.614 to 21.657]	0.287
Weekly weight gain in third trimester (kg)	−17.483	[−85.980 to 51.014]	0.607
Gestationalweightgain (kg)			
Pre-gestational BMI (kg/m^2^)	−0.383	[−0.726 to −0.40]	0.03
Maternal age (years)	0.351	[0.042–0.66]	0.027
EV from UPF in third trimester (kcal)	0.011	[0.004–0.019]	0.006
Parity	0.201	[−1.309 to 1.710]	0.787
Insulin dose in first trimester (UI/kg)	−0.923	[−8.587 to 6.741]	0.806
Insulin dose in second trimester (UI/kg)	−1.451	[−13.277 to 10.376]	0.803
Insulin dose in third trimester (UI/kg)	0.448	[−8.325 to 9.221]	0.917

**Note:**

EV, energetic value; UPF, ultra-processed food; BMI, body mass index.

The variables (*p* < 0.20) related to 1-h postprandial glucose in the bivariate linear regression were: EV consumed from ultra-processed foods in the third trimester of gestation (*p* = 0.008); maternal age (*p* = 0.142); and number of prenatal consultations (*p* = 0.058). Although the *p*-value was >0.20 for weekly weight gain in the third trimester (*p* = 0.406) and number of visits with a nutritionist (*p* = 0.441), they were included in the final model by biological plausibility. The adjusted final model is shown in Table 3. Every 1 kcal in EV from ultra-processed foods in the third trimester raised 1-h postprandial glucose by 0.14 mg/dL in the third trimester (β = 0.143; *p* = 0.011) and each increase in prenatal visits yielded an 8 mg/dL reduction (β = −8.00; *p* = 0.035) in 1-h postprandial glucose in the third trimester. Postprandial 1-h glucose in the second trimester was not influenced by second-trimester ultra-processed food intake (*p* = 0.875).

The same table shows the results of the linear regression referring to total weight gain. The variables tested in the bivariate and related linear regression (*p* < 0.20) were: EV from the third trimester (*p* = 0.002) and pre-gestational BMI (*p* = 0.20). Maternal age at birth (*p* = 0.7), parity (*p* = 0.48), and insulin doses in the first (*p* = 0.90), second (*p* = 0.39), and third (*p* = 0.32) trimesters were also inserted in the multivariate model. In the final adjusted model, every 1 kcal increase in EV from ultra-processed foods in the third trimester increased total gestational weight gain by 0.11 kg (β = 0.11, *p* = 0.006); for each year of maternal age, there was a 0.35 kg increase in gestational weight gain (β = 0.351; *p* = 0.027); and for each 1 kg/m^2^ increase in pre-gestational BMI, there was a 0.38 kg reduction in total weight gain (β = −0.383, *p* = 0.03). Total gestational weight gain was not influenced by the consumption of ultra-processed foods in the second trimester (*p* = 0.66).

## Discussion

In this study, pregnant women with preexisting DM consumed significant amounts of ultra-processed foods, despite regular monitoring by nutritionists during prenatal care. It was found that this consumption was associated with increased maternal blood glucose and gestational weight gain.

There is a shortage of data in the literature regarding consumption of ultra-processed foods by pregnant women and its potential repercussions, especially on women diagnosed with DM, for which comparisons with other population groups would be necessary. Research conducted with the Brazilian population presents similar results regarding the contribution of ultra-processed foods to daily energy consumption, indicating the replacement of traditional dietary patterns based on fresh and minimally processed foods with packaged and ready-to-eat products (Monteiro et al., 2016).

An investigation by Martins et al. (2013) of trends in the proportion of processed foods in total food purchases made by Brazilian households between 1987–1988 and 2008–2009 found a significant increase in the proportion of calories from processed foods to total calories purchased. Indeed, while there was a 10.9% increase in the calorie intake of ultra-processed foods, a significant decline was observed in fresh and minimally processed food consumption. Although this trend applies to households in all income brackets, it was found to be more significant in the lower strata.

Also supporting the findings of the present study, a Brazilian cross-sectional study aimed at evaluating the impact of ultra-processed food consumption on the nutritional profile of diets found that these items accounted for 21.5% of total energy consumption (Louzada et al., 2015).

While Alves-Santos et al. (2016) observed a reduction in the energy contribution of ultra-processed foods to the diet of healthy pregnant women (*n* = 189) compared to the pre-gestational period, such items still represented 41.3% of the diet. The limited changes seen in the dietary patterns of the women indicate that they need to be given more nutritional counseling.

In a study of the diets of women from a poor community of Rio de Janeiro, Brazil, during and after pregnancy, Baião and Deslandes (Baião & Deslandes, 2010) found that they reinterpreted nutritional guidelines and information according to their social and living conditions and experiences. Although “healthy, low-fat, or natural food” is considered by women to be the most appropriate for pregnant women, it was hardly mentioned in their diets. Rather, junk food that basically consisted of ultra-processed food was their preference, rather than “real” food, as it was more practical, cheaper, and more appealing.

In a qualitative descriptive study designed to observe the experiences of pregnant women with DM, Santos et al. (2014) observed that gestation resulted in improvements in control motivated by maternal responsibility. The participants’ concern for their child’s well-being was a milestone for starting to introduce changes, even if they were still hard-won and limited, including their dietary habits.

In our study, eating practices prior to gestation were not evaluated, so there is the possibility that even though the subjects’ ultra-processed food consumption may seem high, it could be lower than in the pre-gestational period. In addition, it is important to consider that eating habits are built throughout a lifetime and are not driven only by physiological needs, but also by external factors such as availability, accessibility, quality, and price, so change may be hard for some individuals (Santos et al., 2014; Claro et al., 2016). An adult population analyzed in a qualitative study described more factors facilitating consumption of ultra-processed foods than barriers, including their flavor, their low cost, addiction, and convenience (Almeida et al., 2018).

The media is also capable of affecting eating habits and perceptions of food. The main products promoted by the food industry in the media are food and beverages that, according to a study conducted in Brazil, are more than 60% ultra-processed foods (Maia et al., 2017). Marketing strategies which in the past focused mainly on television are now often focused on social media, promoting greater interactivity with consumers and thereby achieving a greater power of persuasion in all the age groups (Horta, Rodrigues & Santos, 2018).

Interestingly, for the Brazilian population, preparing food at home is still more affordable than buying processed foods. Claro et al. (2016) show that the average cost of ultra-processed foods is higher than that of other food groups, regardless of the income stratum, geographical area, or urbanization process.

Diet is one of the pillars of DM treatment, and the findings show that dietary planning using carbohydrate counting confers flexibility and greater autonomy to food choices, including for pregnant women, contributing to improved quality of life and glycemic control, with reduction of HbA1c (The DCCT Research Group, 1993; Ulahannan, Ross & Davies, 2007; Laurenzi et al., 2011). However, to achieve such benefits, it is essential to adopt healthy eating habits, especially when considering the high consumption of ultra-processed foods by the Brazilian population, as previously described.

Because of their high energy density and their limited nutritional properties, which promote their excessive consumption, ultra-processed foods are the main food cause associated with weight gain and the development of chronic diseases (Monteiro et al., 2013).

A cross-sectional study using data from the Family Budgets Survey, with a representative sample of the Brazilian population of all age groups, found a daily average consumption of 386 kcal (25.5%) from ultra-processed foods. It also identified an association between an increased prevalence of overweight and obesity and an increase in the calorie contribution of ultra-processed foods in the diet (Canella et al., 2014).

The relationship between weight gain and EV from ultra-processed foods by pregnant women is an important finding, highlighting the contribution of diet to this outcome, especially considering that, due to the high energy density of these foods, it is possible to achieve a high calorie value even from consuming small portions. Excessive weight gain during gestation leads to a number of risks, including: inadequate glycemic control, hypertensive pregnancy syndromes, cesarean delivery, postpartum weight retention, and macrosomia and obesity in the newborn (Goldstein et al., 2017).

A study of women with pre-gestational DM1 has pointed to the risk of newborns being large for gestational age when their mothers gain weight above the recommended level, irrespective of their initial nutritional status, especially in the first trimester. The mean HbA1c was also higher when there was excessive weight gain, with a significant difference at the beginning of gestation, compared to pregnant women with adequate weight gain (Scifres et al., 2014). An increased risk of adverse perinatal outcomes has also been found in pregnant women with DM2 with above-adequate weight gain (Yee et al., 2011). Therefore, it is important to adopt strategies that promote weight gain within the range appropriate for each woman, with a diet based on fresh and minimally processed foods.

When evaluating the contribution of ultra-processed foods to the overall diet of pregnant women (*n* = 45), a study carried out in the United States found that 54.4% of the daily calorie intake (as reported by a food frequency questionnaire) came from this category of foods. As in the present study, the women’s mean total weight gain was 12 kg, and a strong positive association was observed between this gain and a high daily intake of ultra-processed foods, also found for the body fat percentage of the neonate. However, no association with glycemic control was reported (Rohatgi et al., 2017).

There are many factors that influence the decision of consumers to include ultra-processed foods frequently in their diet, including those with access to information about the losses associated with this consumption—as in the case of pregnant women in the present study, where, even at risk of causing unfavorable outcomes for the fetus, in addition to the clinical condition prior to pregnancy, a high intake of this food group was observed.

Among these reasons, it is possible to mention characteristics of ultra-processed products, such as color, aroma and hypers taste resulting from the use of additives; large-scale production; longer shelf life; attractive packaging with health claim; persuasive marketing strategies; and lack of market regulation (Ministério da Saúde, 2014; Monteiro et al., 2013; Pan American Health Organization (PAHO), 2015). Studies also point to the modification of consumers’ shopping environments—from fairs, produce and small markets to supermarkets and fast food chains—as facilitators for the acquisition of ultra-processed products (Monteiro et al., 2013; Pan American Health Organization–PAHO, 2015).

In addition, nutritional guidelines recommended by trained health professionals, potential strategies to reduce the consumption of ultra-processed products refer to the design and implementation of more effective public policies (Swinburn et al., 2019). Evidence indicates the need for greater market regulation actions and fiscal measures that favor the large food, production, and sales industries less, with an increase in taxes on these products; tax incentives to strengthen family farming and small producers; creation of community environments that facilitate healthy eating practices; restriction of marketing in public spaces; and clear and informative alerts regarding nutritional composition on the front of industrialized product labels (Pan American Health Organization–PAHO, 2015; Swinburn et al., 2019; Corvalán et al., 2018).

One of the limitations of the present study is that the consultation protocol and the database were developed prior to the publication of the new Food Guide for the Brazilian Population, which means the consumption of ultra-processed foods may have been underreported. The small sample size may also be a limiting factor. Considering that DM in pregnancy is still relatively rare, even though it is increasing, it is hard to find a large number of women at the same health unit with the condition prior to pregnancy.

One positive factor of the study is the presence of significant findings regarding ultra-processed food consumption and adverse perinatal outcomes, even with a small number of pregnant women. The analysis of ultra-processed food consumption by pregnant women with pre-existing DM is also a novel aspect of the study, making important contributions to the nutritional care of these women.

## Conclusions

The results indicate the significant consumption of ultra-processed foods by pregnant women with pre-existing DM using carbohydrate counting, which is consistent with dietary habits amongst the Brazilian population in general. The association between calorie intake from these foods and HbA1c, postprandial blood glucose, and total weight gain exposes the risks posed by inadequate nutrition, which may affect maternal health and fetal development.

Given that pre-gestational DM predisposes women to an increased risk of adverse perinatal outcomes, reducing the intake of ultra-processed foods in the diet could contribute to a better prognosis in the short and long term. The findings reinforce that the importance of the consumption of minimally processed, fresh, home-cooked food, rather than processed foods by pregnant women and their families should be reinforced at each consultation and through food, nutritional, and health education actions.

## Supplemental Information

10.7717/peerj.10514/supp-1 Supplemental Information 1 GMD Dataset.

## Additional Information and Declarations

### Data Availability

The following information was supplied regarding data availability: The raw measurements are available as a Supplemental File.
